# Balloon-Expandable TAVR Bioprostheses: Area or Perimeter Sizing? A Prospective Pilot Study

**DOI:** 10.1155/2022/3139476

**Published:** 2022-10-18

**Authors:** Jonathan Halim, Peter den Heijer, Jeroen Vos, Bas E. Schölzel, Martijn Meuwissen, Ben van den Branden, Andreas Baumbach, Alexander J. J. Ijsselmuiden

**Affiliations:** ^1^Department of Cardiology, Amphia Hospital Breda, Molengracht 21, 4818 CK, Breda, Netherlands; ^2^Queen Mary University of London, Barts Heart Centre, London, UK

## Abstract

**Objective:**

In TAVR, area sizing is used for balloon-expandable (BE) valves, whereas self-expanding valves are sized to annulus perimeter. For BE valves, this seems illogical: these frames force a circular shape even on an ellipsoid annulus. This can potentially lead to relative undersizing when area sizing is being applied. We developed a perimeter-based sizing algorithm to evaluate the safety and feasibility of perimeter sizing for the Myval BE valve.

**Methods:**

In this prospective single-center study, 60 patients with severe aortic stenosis treated with the Myval BE valve were included. Perimeter sizing was used with limited oversizing of 3.7% ± 1.3% compared to the annulus perimeter. After TAVR, clinical outcomes were evaluated at 30 days and 1 year. An echocardiographic follow-up took place at 30 days.

**Results:**

At 30 days, the need for PPI and stroke occurred in 2% and 3% of the patients, respectively. Moreover, cardiac death and moderate-severe PVL were absent. At 1-year, cardiac death and stroke were observed in 3% and 8% of the patients, respectively. In 33.3% of the patients, a larger valve size was implanted compared to the valve size calculated by area sizing.

**Conclusions:**

Perimeter sizing with the Myval BE valve leads to substantial use of larger valve sizes and favorable clinical outcomes, with low PPI and the absence of significant PVL. A randomized controlled trial is being planned to prove the superiority of this alternative sizing method.

## 1. Introduction

Appropriate device size selection in transcatheter aortic valve replacement (TAVR) is pivotal. An inappropriately undersized bioprosthetic valve is known to be associated with adverse events, such as moderate or severe paravalvular leakage (PVL) and device embolization, while oversizing may carry risks for annular rupture, the need for a permanent pacemaker implantation (PPI), or coronary obstruction [1–6].

**Figure 1:**
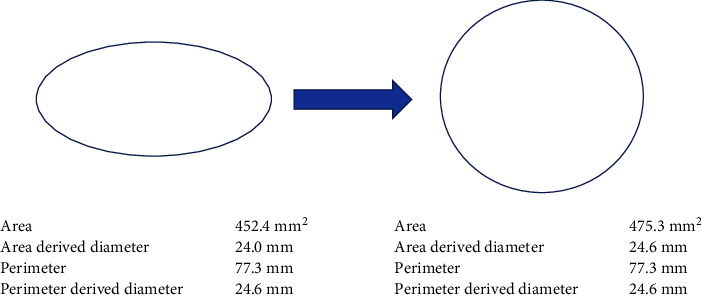
The annulus area in relation to the aortic annulus shape. Schematic drawing of an oval-shaped annulus forced into a circular shape, retaining its perimeter but increasing its area.

In TAVR, manufacturers of balloon-expandable (BE) valves recommend using annulus area to calculate optimal frame size, whereas self-expanding valves, per instructions for use, are usually sized to annulus perimeter [7, 8]. Nevertheless, it seems logical to apply perimeter sizing as well to BE valves. In contrast to the annulus area, the annulus perimeter is not influenced by shape changes. When the severity of the aortic stenosis increases, the aortic annulus will adopt a more oval shape. As a result, the annulus area will reduce disproportionately compared to the annulus perimeter. Self-expanding valves tend to keep the shape of the annulus after delivery and do not change the annulus area or perimeter. In contrast, BE valves force the annulus into a round shape, thereby—in the case of an oval shape-increasing the annulus area relative to its unchanged perimeter after implantation (Figure 1). This can lead to a potential underestimation of the true aortic annulus and the risk of undersizing [9].

The Myval BE valve (Meril Life sciences, Vapi, India) is a novel transcatheter heart valve (THV) system, with its safety and efficacy being confirmed in the MyVal-1 study [10]. A unique property of the Myval BE valve is the availability of intermediate and XL valve sizes, providing a more tailored device size selection to minimize the risk of over or undersizing. The Landmark trial, which is a randomized controlled trial and is currently enrolling patients, will compare the Myval BE valve with contemporary THV systems. [11].

Clinical data on perimeter sizing for BE valves is still lacking. For this reason, the present study sought to evaluate the applicability of perimeter sizing for the Myval BE valve. Our hypothesis is that in a significant part of the patients, a different size BE valve will be selected with perimeter sizing compared to area sizing. Furthermore, we believe that perimeter sizing for the Myval BE valve is a safe method associated with a low rate of serious adverse events.

## 2. Methods

### 2.1. Study Design

In this prospective single-center pilot study, 60 patients undergoing TAVR with the Myval BE valve were included. In these patients, TAVR was performed between August 2020 and July 2021 at the Amphia Hospital in Breda, The Netherlands. All patients had native symptomatic, severe aortic stenosis and were eligible for TAVR after the Heart Team discussion. Bicuspid cases were excluded from this study. Informed consent was obtained in accordance with the Declaration of Helsinki.

Preprocedural planning with a multidetector computed tomography utilizing dedicated software was performed (3mensio, Pie Medical Imaging, Maastricht, The Netherlands). Then, the optimal valve size was calculated using both area and perimeter sizing. For perimeter sizing, we developed a precise perimeter-based sizing algorithm specifically for the Myval BE valve. Using this algorithm, we tried to limit the oversizing of the annulus to a maximum of 5% of its perimeter. Oversizing was defined by the valve frame diameter divided by the annulus perimeter. In all patients, the valve size selection was eventually determined by perimeter sizing and compared to the size based on area.

In the case of borderline anatomy, we often decided to select the smaller device and increase the filling volume to facilitate the full expansion of the balloon. The femoral artery was the preferred access route. However, if a femoral access route was deemed unsuitable, a transapical access route was chosen. All patients received general anesthesia during TAVR, and the femoral artery was approached and closed surgically. Transesophageal echocardiography was used in all patients to optimize TAVR results. We aimed for absence, trace, or at least mild PVL by determining with transesophageal echocardiography whether postdilatation with an increased balloon volume was deemed necessary. After TAVR, clinical evaluation took place at 30 days and 1 year. An echocardiographic follow-up took place at 30 days.

### 2.2. Myval THV System

The frame of the Myval BE valve consists of nickel-cobalt alloy [12]. It has a hybrid honeycomb structure, with the upper 53% of the frame having large open cells, in order to preserve coronary flow, and the lower 47% of the frame having closed cells, resulting in higher radial strength. The lower closed cells are covered with a polyethylene terephthalate cuff to minimize PVL. A decellularized bovine pericardium tri-leaflet valve is present with additional anticalcification treatment (AntiCa™, Meril Life Sciences, Vapi, India), which is fixed with glutaraldehyde at the site.

Besides the traditional sizes (20, 23, 26, and 29 mm), intermediate sizes (21.5, 24.5, and 27.5 mm) and extra-large sizes (30.5 and 32 mm) are also available for the Myval BE valve. Furthermore, slightly changing implantation diameters by adding or subtracting 1-2 ml of indeflator saline/contrast, decreases these steps to 0.5 mm, compared to the 3 mm steps of other BE or self-expanding valves.

A 14 Fr Python sheath is utilized for THV delivery. Before vessel insertion of the Navigator balloon-catheter delivery system, the THV is crimped on the delivery system. The delivery system has a balloon located distally with two internal expansion ports, resulting in simultaneous expansion proximally and distally. Importantly, the THV is deployed at the annular position, and positioning at this level is facilitated by the alternative dark-light band-like pattern. Lastly, the THV can be retrieved in the 14 Fr Python sheath if undelivered.

### 2.3. Study Endpoints

Clinical outcomes were evaluated at 30 days and 1 year using Valve Academic Research Consortium-2 criteria. All-cause death, cardiac death, stroke, myocardial infarction, acute kidney injury (stage 2 or 3), access-site related vascular and bleeding complications, moderate or severe PVL, conduction system disturbances resulting in new PPI, and whether a larger valve was selected by means of perimeter sizing than would have been chosen by area sizing were investigated. Echocardiographic outcomes were assessed at 30 days.

### 2.4. Statistical Analysis

All continuous variables are expressed as a mean and standard deviation. Categorical variables are presented as frequency and percentage. All analyses were conducted with SPSS v.26 (IBM, Chicago, IL, USA).

## 3. Results

### 3.1. Baseline Characteristics

60 patients who received a Myval BE valve were included in our study. The baseline characteristics of these patients are shown in Table 1. The mean age in this group of patients was 80.2 ± 6.6 years, with 50% of the patients being men. The mean Euroscore II was 4.0 ± 2.8 with moderate-severe left ventricle dysfunction observed in 15% of the patients. A pacemaker had been previously implanted in 8% of the patients. The mean annulus perimeter and mean annulus area were 78.3 ± 7.4 mm and 469.5 ± 83.7 mm^2^, respectively.

### 3.2. Procedural Outcomes

**Figure 2:**
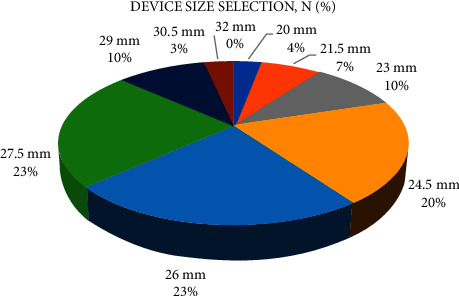
Device size selection.

The femoral artery was the access site of choice in 83% of the patients. For the rest of the patients (17%), a transapical TAVR was performed. Predilatation was performed in 3% of the patients, whereas none received postdilatation. An overview of the device size selection is illustrated in Figure 2.

In our study population, 20 patients (33.3%) received a valve size that was one size larger than what would have been chosen when area sizing was used as a sizing parameter. In this group, despite using larger sizes in one third of the patients, with our sizing algorithm we limited perimeter oversizing to only 3.7 ± 1.3%. Moreover, a higher eccentricity index could be observed in the patients receiving a larger valve size (*n* = 20) compared to the patients (*n* = 40) who received the same valve size if area sizing would have been applied (0.26 vs. 0.21, *p*=0.002).

### 3.3. Clinical Outcomes

Clinical outcomes are summarized in Table 2. Procedure-related adverse events such as coronary obstruction, annular rupture, or valve embolization were not observed. At 30 days, death and the presence of a myocardial infarction were not seen in our study population. A PPI was needed in one patient (2%) due to a high-grade AV block after TAVR. Stroke and acute kidney injury both occurred in two patients (3%). Importantly, complete recovery of kidney function took place in both patients during hospitalization, indicating a prerenal cause of the kidney injury. Major access-site-related vascular and major bleeding complications were absent.

Between 30-day and 1-year follow-up, all-cause death was present in six patients (10%). In two of these six patients, end-stage heart failure leading to death was documented. The other four patients died due to a noncardiac-related cause. Moreover, stroke was observed in three patients (5%). Other major adverse events were absent at the 1-year follow up.

### 3.4. Echocardiographic Outcomes

**Figure 3:**
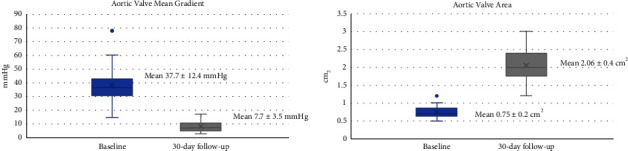
Aortic valve hemodynamics at 30 days. Abbreviations: TAVR = Transcatheter aortic valve replacement.

At 30 days, moderate-severe PVL was absent in all patients. Improved valve hemodynamics could be observed after TAVR with an increase in the aortic valve area from 0.75 ± 0.2 cm^2^ to 2.06 ± 0.4 cm^2^ and a decrease in the aortic valve mean gradient from 37.7 ± 12.4 mm·Hg to 7.7 ± 3.5 mm·Hg (Figure 3).

## 4. Discussion

In this prospective single-center study, perimeter sizing with the Myval BE valve has been assessed, and to our knowledge, this is the first study evaluating the safety and feasibility of perimeter sizing for BE valves. The main findings of this study are that: (1) perimeter sizing for the Myval BE valve is safe and is associated with a low rate of PPI and absence of moderate-severe PVL; (2) favorable 1-year clinical outcomes are observed with the Myval BE valve; and (3) perimeter sizing leads to a larger valve size selection in a significant part of the patients.

Traditionally, area sizing is used for BE valves, and it is known to be associated with good clinical outcomes and a low risk for adverse events, such as moderate-severe PVL, annular rupture, and the need for PPI. [3, 5, 13, 14].

Nevertheless, it can be debated whether or not perimeter sizing should be preferred over area sizing in BE valves. The aortic annulus perimeter has the advantage of showing minimal variation during the cardiac cycle, and in contrast to the annulus area, it is not affected by shape changes. So, when the aortic annulus becomes more ellipsoid with the progression of the aortic stenosis, the aortic annulus perimeter will remain stable, while the annulus area will reduce disproportionately [9, 15]. In the study of Blanke et al., both the aortic annulus perimeter and the annulus area were able to predict PVL to a similar extent in BE valves [16]. This finding was confirmed in our study by the absence of moderate-severe PVL in our study population.

Nevertheless, using different measures for annulus sizing and taking into account the more pronounced aortic annulus eccentricity in severe aortic stenosis can have implications on device size selection. Hence, it can be expected that a smaller device size is more often selected with area sizing, with a potential risk of undersizing. This was also seen in our study, with a larger valve size being implanted in the perimeter sizing group in 33.3% of the patients (*n* = 20) than calculated by area sizing. Importantly, in these 20 patients, the eccentricity index was higher compared to the other patients in the perimeter sizing group. Consequently, a larger percentage increase of an aortic valve area after TAVR could be observed in our study population compared to earlier studies where area sizing was used [13, 14, 17]. These findings confirm that area sizing in patients with a flat, ellipsoid annulus will often result in undersizing.

In addition, selecting a larger valve size in a significant part of the patients than calculated by area sizing appears to be safe with a low rate of PPI and absence of annular rupture or coronary obstruction in our study population.

We can therefore conclude that perimeter sizing may be a promising sizing method for the Myval BE valve. Additionally, under-or overfilling the balloon by subtracting or adding 1-2 ml of indeflator saline/contrast is another technique that can accommodate an optimal device size implantation for the Myval BE valve. We believe that it is crucial to determine the most optimal sizing strategy for BE valves in order to improve clinical outcomes in an increasingly larger and younger target population for TAVR. Based on the outcome of this pilot study, we are planning an investigator-initiated, randomized controlled trial to further elucidate if perimeter sizing for the Myval BE valve is the most optimal sizing strategy compared to area sizing.

## 5. Limitations

This study has several limitations. First, the relatively small sample size and the fact that it is an observational study with known limitations. Consequently, a comparison cannot be made between area and perimeter sizing. Furthermore, a relatively short follow-up period was implemented for this study, with no available information on long-term clinical outcomes with perimeter sizing.

## 6. Conclusion

Perimeter sizing for the Myval BE valve is safe and feasible. A substantial use of larger valve sizes, a low rate of PPI, and no significant PVL have been observed. A randomized controlled trial will be conducted to assess which sizing method is superior.

## Figures and Tables

**Table 1:** Baseline characteristics.

*n* = 60 *N* (%) or mean ± SD	
*Patient characteristics*	
Age	80.2 ± 6.6
Male	30 (50)
BMI	28.6 ± 4.8
Euroscore II	4.0 ± 2.8
NYHA class III or IV	23 (38)
Diabetes mellitus	26 (43)
Hypertension	45 (75)
Coronary artery disease	32 (53)
Previous CABG	7 (12)
Chronic kidney disease	23 (38)
Cerebrovascular disease	11 (18)
Peripheral vascular disease	11 (18)
COPD	9 (15)
Atrial fibrillation	23 (38)
Prior pacemaker	5 (8)
RBBB	7 (12)
LBBB	5 (8)

*Echocardiographic measurements*	
LVEF≤ 40%	9 (15)
AV area, (cm^2^)	0.77 ± 0.2
AV mean gradient, (mm·Hg)	37.1 ± 12.8
Moderate or severe mitral regurgitation	8 (13)

*MDCT measurements*	
Annulus perimeter, (mm)	78.3 ± 7.4
Annulus area, (mm^2^)	469.5 ± 83.7
Maximum annulus diameter, (mm)	27.4 ± 4.7
Mean annulus diameter, (mm)	24.5 ± 2.3
Minimum annulus diameter, (mm)	21.3 ± 2.1
Minimum femoral artery diameter, (mm)	6.3 ± 1.3

BMI = Body mass index, NYHA = New york heart association, CABG = Coronary artery bypass grafting, COPD = Chronic obstructive pulmonary disease, RBBB = Right bundle branch block, LBBB = Left bundle branch block, LVEF = Left ventricular ejection fraction, AV = Aortic valve, MDCT = Multidetector computer tomography.

**Table 2:** Clinical outcomes.

*n* = 60 *N* (%)	30 days	1 year
All-cause death	0 (0)	6 (10)
Cardiac death	0 (0)	2 (3)
Myocardial infarction	0 (0)	0 (0)
PPI	1 (2)	1 (2)
Moderate-severe PVL	0 (0)	—
Stroke	2 (3)	5 (8)
Acute kidney injury (stage 2 or 3)	2 (3)	—
Vascular complication	0 (0)	—
Minor bleeding	2 (3)	—
Major/Life-threatening bleeding	0 (0)	—

PPI = Permanent pacemaker implantation, PVL = Paravalvular leakage.

## Data Availability

The data used to support the findings of this study are available from the corresponding author upon reasonable request.

## Abbreviations

TAVR: Transcatheter aortic valve replacement
PVL: Paravalvular leakage
PPI: Permanent pacemaker implantation
BE: Balloon-expandable
THV: Transcatheter heart valve.
